# A case report of a novel 22 bp duplication within exon 1 of the UGT1A1 in a Sudanese infant with Crigler-Najjar syndrome type I

**DOI:** 10.1186/s12876-020-01192-4

**Published:** 2020-03-06

**Authors:** Sailaja Valmiki, Kiran Kumar Mandapati, Leela Krishna Vamsee Miriyala, Chayarani Chandrashekhar Kelgeri, Mohamed Rela, Naresh P. Shanmugam, Durga Rao Vegulada

**Affiliations:** 1 Department of Molecular Diagnostics, Genes N Life Healthcare Pvt. Ltd., Punjagutta, Hyderabad, 500 082 India; 2 Department of Paediatric Gastroenterology, Hepatology and Transplantation, Institute of Liver Diseases and Transplantation, Gleneageles Global Health City, Chennai, India

**Keywords:** Crigler-Najjar syndrome, Autosomal recessive, Bilirubin, UGT1A1, Mutation, Liver transplant

## Abstract

**Background:**

Crigler Najjar type 1 is a rare autosomal recessive condition caused by the absence of UDPGT enzyme due to mutations in the UGT1A1 gene. This enzyme is responsible for elimination of unconjugated bilirubin from the body by glucuronidation. Affected individuals are at risk for kernicterus and require lifelong phototherapy. Liver transplant is the only definitive treatment.

**Case presentation:**

Here we report a case of a 6 month old Sudanese female infant with CN1 whose molecular analysis revealed a novel homozygous 22 base pair duplication (c.55_76dup) in the coding exon 1 of the UGT1A1 gene. This 22 bp duplication causes a frame shift leading to a premature stop codon. She underwent a successful liver transplant at 7 months of age and is doing well at 1 year follow-up.

**Conclusion:**

This study shows that molecular diagnosis helps in precise diagnosis of CN1 and in prognosis, prompt medical intervention and appropriate therapy. This particular 22 bp duplication within the coding region of UGT1A1 can be a founder mutation in the Sudanese population.

## Background

Crigler-Najjar syndrome type I (CN1, MIM#218800), Crigler-Najjar syndrome type II (CN2, MIM# 606785), and Gilbert syndrome (GS, MIM# 143500) are a group of hereditary unconjugated hyperbilirubinemias resulting from mutations within the UDP glucuronosyltransferase family 1 member A1 (UGT1A1) gene, located on chromosome 2q37 locus [1]. It consists of 5 exons and is expressed in the liver, colon, intestine and stomach [2, 3]. The liver UGT1A1 is the sole enzyme responsible for the bilirubin metabolism [3]. To date, more than 110 mutations within UGT1A1 gene have been reported in the Human Gene Mutation Database [4]. The genetic alterations reported are in the UGT1A1 promoter and all five UGT1A1 exons [5].

Crigler Najjar type 1 (CN1) is inherited as an autosomal recessive disorder of bilirubin conjugation. It presents shortly after birth and is characterized by complete or near complete absence of the UGT1A1 enzyme activity. In recent years molecular characterization has become an essential component in differentiating CN1, CN2 and GS [3]. The pathogenesis of GS can largely be explained by TA insertion polymorphism in the UGT1A1 promoter region which causes reduced expression of the UGT1A1 enzyme [6]. Missense/nonsense mutations in UGT1A1 gene accounts for large numbers of CN1 and CN2 cases [7]. Homozygous or compound heterozygous mutations resulting in some residual UGT1A1 enzyme activity cause CN2, whereas, complete absence of bilirubin glucuronidation and removal cause CN1 [3, 8]. Here we report a novel homozygous 22 base pair duplication in the coding exon 1 of the UGT1A1 gene in a Sudanese infant resulting in CN1 who subsequently underwent successful liver transplant.

The study was approved by institutional ethical committee of the Genes N Life Health Care private limited, Hyderabad. Written informed consent was obtained from the patient’s parents.

### Case presentation

A 6 month old Sudanese female infant with a clinical diagnosis of Crigler Najjar Syndrome Type 1 (CN1) was referred for consideration of liver transplant. She was born to second degree consanguineous parents. The pregnancy was uncomplicated and she was delivered at term by lower segment caesarean section weighing 2.5 Kg. She was noticed to be jaundiced at birth and phototherapy was initiated. The diagnosis of CN1 became apparent in a couple of weeks in view of persistent unconjugated Hyperbilirubinemia. Her unconjugated bilirubin ranged between 20 and 25 mg/dl, with conjugated fraction of less than 0.6 mg/dl. There was no evidence of haemolysis or liver dysfunction. The infant was exclusively breast fed and was developing appropriately for her age. The child was receiving home phototherapy every day at the time of referral. After admitting into our hospital we put her on standard 10 to 12 h of phototherapy every day.

Clinical examination revealed her to be icteric with normal systemic examination. Her weight and height were 6.9 kg and 65 cms respectively, both at 50 centile. Her development and neurology was appropriate for age. Apart from elevated unconjugated bilirubin, she had unremarkable full blood count, liver transaminases, coagulation profile, serum albumin and liver radio imaging(We performed liver ultrasound Doppler and CT abdomen with contrast as per the protocol).

Standard procedures were employed to obtain genomic DNA from the index case and her parents. The promoter region and all the five coding exons with the intron exon boundaries were PCR amplified. All the PCR products were purified and Sanger sequenced. Bidirectional sequencing was carried out and subjected to sequence analysis. PCR conditions and primer pair details are presented in Table 1.

**Table 1:** *UGT1A1* gene primers used in PCR and Sequencing

Region	Primer Name	Primer sequence	Annealing Temp.	Amplicon size bp
Promoter	UGT1A1- P-F	AACTCCCTGCTACCTTTGTGGA	60	473
	UGT1A1- P-R	TGATCACACGCTGCAGGAAAGA		
Exon1	UGT1A1-E1A-F	AGGAGCAAAGGCGCCATGGCT	60	528
	UGT1A1-E1A-R	GAAGAATACAGTGGGCAGAGAC		
	UGT1A1-E1B-F	CATGCTGACGGACCCTTT	54	588
	UGT1A1-E1B-R	GATGCCAAAGACAGACTCAAAC		
Exon 2	UGT1A1-E2-F	CTCAAACACGCATGCCTTTAAT	54	322
	UGT1A1-E2-R	GAAGCTGGAAGTCTGGGATTAG		
Exon 3	UGT1A1-E3-F	AAGACTGTTCCTTCAGAGGAC	58	283
	UGT1A1-E3-R	AGCTCAACAATCCTTTAGAATAGC		
Exon 4	UGT1A1-E4-F	TGCAAGGGCATGTGAGTAA	56	432
	UGT1A1-E4-R	GAAACAACGCTATTAAATGCTAG		
Exon 5	UGT1A1-E5-F	GCCATGAGCATAAAGAGAGGAT	60	597
	UGT1A1-E5-R	CCTGATCAAAGACACCAGAGG		

PCR conditions: PCR reaction mixture (10 μL) contained 1 µL of genomic DNA, 0.25 μL of each forward and reverse primer (diluted to 10 μmol/L), 3.5 μL of ddH2O, 5 μL of 2 × Taq PCR Master Mix (Emerald GT mix, Clontech). PCR was implemented by denaturation at 94 °C for 10 s, followed by 40 thermal cycles composed of 98 °C for 30 s, 60 °C(promoter) for 30 s, and 72 °C for 1 min, with a final extension at 72 °C for 10 min

**Fig. 1:**
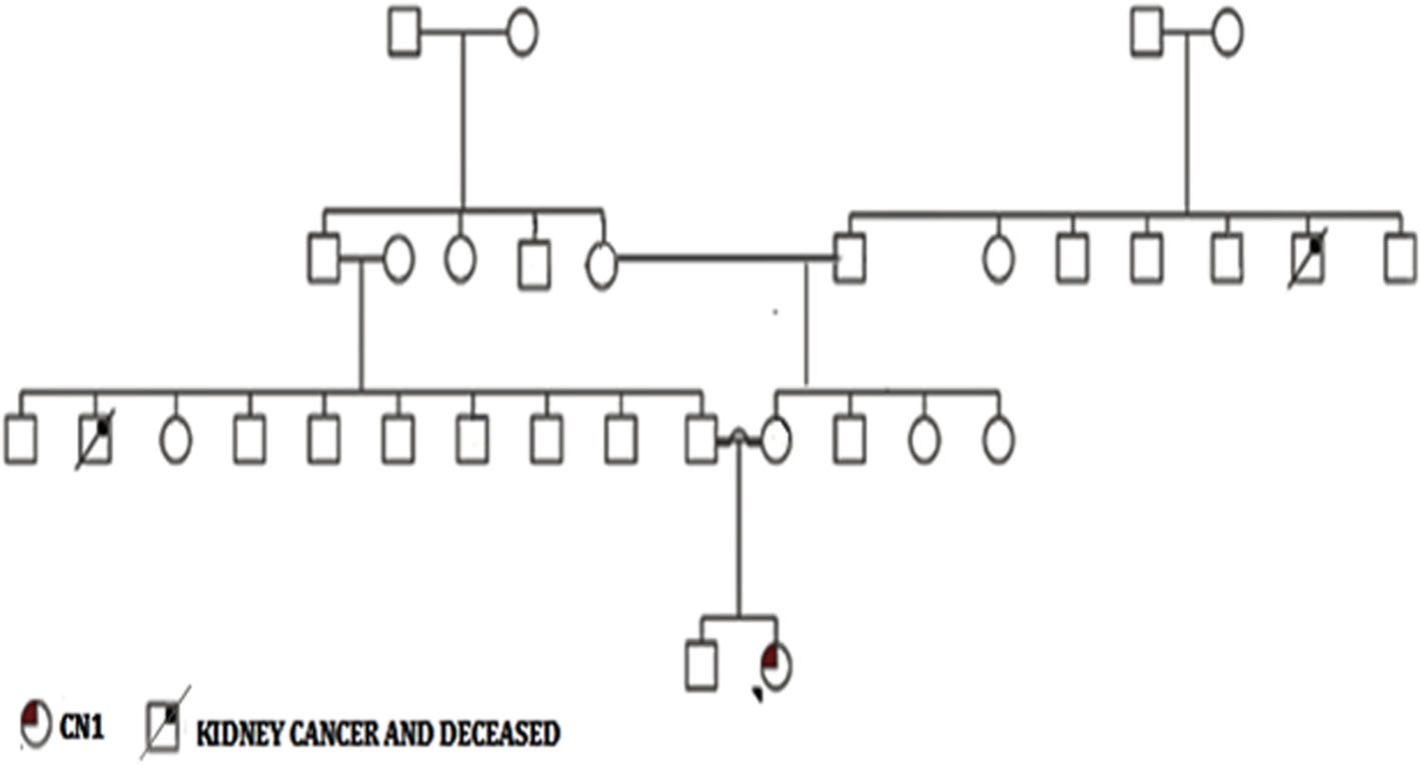
Detailed Pedigree of the family

**Fig. 2:**
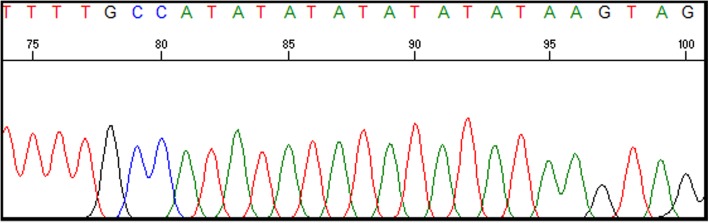
Normal wild type TATA box (A(TA)6TAA)sequence

**Fig. 3:**
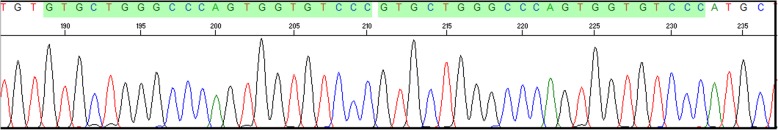
A novel homozygous 22 bp duplication (c.55_76dup) in the coding region of the Exon1

A detailed pedigree of the family was recorded (Fig. 1). Our patient was born to second degree consanguineous parents and there was no other case of CN1 reported in all the three generations. UGT1A1 full gene analysis was performed using polymerase chain reaction (PCR) followed by Sanger DNA sequencing. Molecular analysis revealed a normal wild type TATA box (A(TA)6TAA)sequence in our patient and her parents (Fig. 2). Further analysis of the exon-intron boundaries in the patient revealed a novel homozygous 22 bp duplication (c.55_76dup) in the coding region of the Exon1 (Fig. 3). This 22 bp duplication causes a frame shift leading to a premature stop codon resulting in a truncated 62 amino acids protein. Both parents of the patient were heterozygous for the 22 bp (c.55_76dup) duplication, indicating a clear recessive pattern of inheritance. No mutations were detected in the other coding exons.

Our patient subsequently received a living related left lateral liver segment from her father. She had a smooth intraoperative period and her postoperative period was unremarkable except for 2 episodes of biopsy proven acute cellular rejection which responded to steroids. Her immunosuppression was performed with Tacrolimus, Mycophenolate mofetil and low dose steroids. Post transplantation bilirubin levels normalized, unconjugated bilirubin levels were found to be 0.8 mg/dL and conjugated bilirubin was 0.3 mg/dL. Post-surgical monitoring of the infant was done with shared care between our team of doctors and Sudanese doctors who took care of her locally. She periodically got blood tests done with immunosuppression levels which are sent to us electronically for advice. She is currently well at 1 year follow up.

## Discussion and conclusion

Mutations in the *UGT1A1* gene were previously shown to cause CNS. The novel homozygous 22 bp duplication (c.55_76dup) causes frame shift in the coding sequence, leading to premature stop codon and resulting in a truncated non-functional UGT1A1 enzyme or a complete loss of production of the bilirubin UDP-glucuronosyltransferase (UDPGT). UGT1A1 catalyzes the glucuronidation of 7-ethyl-10-hydroxycamptothecin (SN-38), the active metabolite of irinotecan and endobiotic compounds like bilirubin. Mutations leading to complete loss of function of UGT1A1 accumulate the toxic metabolites and unconjugated bilirubin leading to hyperbilirubinemia, jaundice, and sometimes, kernicterus leading to death.

The Human Gene Mutation Database [5] search as of January, 2019 shows no duplication mutation(s) causing CN1. This particular 22 bp genetic alteration could be a founder mutation in Sudanese population. We recommend screening for this novel mutation in Sudanese patients with unconjugated hyper bilirubinemias to confirm the founder status of the mutation.

Parents in this case were heterozygous for the 22 bp duplication with wild type (A(TA)6TAA) promoter polymorphism and the presence of wild type rules out GS. Our patient had inherited two copies of the 22 bp duplication alleles which might have resulted in severe phenotype.

In conclusion, we report a case of CN1 resulting from a novel 22 bp duplication within the coding region of UGT1A1 in a Sudanese infant. Our findings will help in understanding the genetic susceptibility to CN1 in Sudanese descent with unconjugated hyperbilirubinemia. Prompt diagnosis and medical intervention followed by living related donor liver transplant helped to save the baby and also prevented from kernicterus.

## Data Availability

Data sharing is not applicable to this article as no datasets were generated. Molecular sequences are available with the corresponding author which can be shared on reasonable request.

## Abbreviations

CN1: Crigler Najjar syndrome type I
UGT1A1: UDP glucuronosyltransferase family 1 member A1
